# Publisher Correction: Population genomics of post-glacial western Eurasia

**DOI:** 10.1038/s41586-024-07044-5

**Published:** 2024-01-18

**Authors:** Morten E. Allentoft, Martin Sikora, Alba Refoyo-Martínez, Evan K. Irving-Pease, Anders Fischer, William Barrie, Andrés Ingason, Jesper Stenderup, Karl-Göran Sjögren, Alice Pearson, Bárbara Sousa da Mota, Bettina Schulz Paulsson, Alma Halgren, Ruairidh Macleod, Marie Louise Schjellerup Jørkov, Fabrice Demeter, Lasse Sørensen, Poul Otto Nielsen, Rasmus A. Henriksen, Tharsika Vimala, Hugh McColl, Ashot Margaryan, Melissa Ilardo, Andrew Vaughn, Morten Fischer Mortensen, Anne Birgitte Nielsen, Mikkel Ulfeldt Hede, Niels Nørkjær Johannsen, Peter Rasmussen, Lasse Vinner, Gabriel Renaud, Aaron Stern, Theis Zetner Trolle Jensen, Gabriele Scorrano, Hannes Schroeder, Per Lysdahl, Abigail Daisy Ramsøe, Andrei Skorobogatov, Andrew Joseph Schork, Anders Rosengren, Anthony Ruter, Alan Outram, Aleksey A. Timoshenko, Alexandra Buzhilova, Alfredo Coppa, Alisa Zubova, Ana Maria Silva, Anders J. Hansen, Andrey Gromov, Andrey Logvin, Anne Birgitte Gotfredsen, Bjarne Henning Nielsen, Borja González-Rabanal, Carles Lalueza-Fox, Catriona J. McKenzie, Charleen Gaunitz, Concepción Blasco, Corina Liesau, Cristina Martinez-Labarga, Dmitri V. Pozdnyakov, David Cuenca-Solana, David O. Lordkipanidze, Dmitri En’shin, Domingo C. Salazar-García, T. Douglas Price, Dušan Borić, Elena Kostyleva, Elizaveta V. Veselovskaya, Emma R. Usmanova, Enrico Cappellini, Erik Brinch Petersen, Esben Kannegaard, Francesca Radina, Fulya Eylem Yediay, Henri Duday, Igor Gutiérrez-Zugasti, Ilya Merts, Inna Potekhina, Irina Shevnina, Isin Altinkaya, Jean Guilaine, Jesper Hansen, Joan Emili Aura Tortosa, João Zilhão, Jorge Vega, Kristoffer Buck Pedersen, Krzysztof Tunia, Lei Zhao, Liudmila N. Mylnikova, Lars Larsson, Laure Metz, Levon Yepiskoposyan, Lisbeth Pedersen, Lucia Sarti, Ludovic Orlando, Ludovic Slimak, Lutz Klassen, Malou Blank, Manuel González-Morales, Mara Silvestrini, Maria Vretemark, Marina S. Nesterova, Marina Rykun, Mario Federico Rolfo, Marzena Szmyt, Marcin Przybyła, Mauro Calattini, Mikhail Sablin, Miluše Dobisíková, Morten Meldgaard, Morten Johansen, Natalia Berezina, Nick Card, Nikolai A. Saveliev, Olga Poshekhonova, Olga Rickards, Olga V. Lozovskaya, Olivér Gábor, Otto Christian Uldum, Paola Aurino, Pavel Kosintsev, Patrice Courtaud, Patricia Ríos, Peder Mortensen, Per Lotz, Per Persson, Pernille Bangsgaard, Peter de Barros Damgaard, Peter Vang Petersen, Pilar Prieto Martinez, Piotr Włodarczak, Roman V. Smolyaninov, Rikke Maring, Roberto Menduiña, Ruben Badalyan, Rune Iversen, Ruslan Turin, Sergey Vasilyev, Sidsel Wåhlin, Svetlana Borutskaya, Svetlana Skochina, Søren Anker Sørensen, Søren H. Andersen, Thomas Jørgensen, Yuri B. Serikov, Vyacheslav I. Molodin, Vaclav Smrcka, Victor Merts, Vivek Appadurai, Vyacheslav Moiseyev, Yvonne Magnusson, Kurt H. Kjær, Niels Lynnerup, Daniel J. Lawson, Peter H. Sudmant, Simon Rasmussen, Thorfinn Sand Korneliussen, Richard Durbin, Rasmus Nielsen, Olivier Delaneau, Thomas Werge, Fernando Racimo, Kristian Kristiansen, Eske Willerslev

**Affiliations:** 1 Lundbeck Foundation GeoGenetics Centre, Globe Institute, University of Copenhagen, Copenhagen, Denmark; 2 Trace and Environmental DNA (TrEnD) Laboratory, School of Molecular and Life Sciences, Curtin University, Perth, Western Australia Australia; 3 Department of Historical Studies, University of Gothenburg, Gothenburg, Sweden; 4 Sealand Archaeology, Kalundborg, Denmark; 5 GeoGenetics Group, Department of Zoology, University of Cambridge, Cambridge, UK; 6 Department of Genetics, University of Cambridge, Cambridge, UK; 7 Institute of Biological Psychiatry, Mental Health Services, Copenhagen University Hospital, Roskilde, Denmark; 8 Department of Computational Biology, University of Lausanne, Lausanne, Switzerland; 9 Swiss Institute of Bioinformatics, University of Lausanne, Lausanne, Switzerland; 10 Department of Integrative Biology, University of California, Berkeley, CA USA; 11 Research Department of Genetics, Evolution and Environment, University College London, London, UK; 12 Department of Archaeology, University of Cambridge, Cambridge, UK; 13 Laboratory of Biological Anthropology, Department of Forensic Medicine, University of Copenhagen, Copenhagen, Denmark; 14 Muséum National d’Histoire Naturelle, CNRS, Université de Paris, Musée de l’Homme, Paris, France; 15 The National Museum of Denmark, Copenhagen, Denmark; 16 Section for Evolutionary Genomics, Globe Institute, University of Copenhagen, Copenhagen, Denmark; 17 Centre for Evolutionary Hologenomics, University of Copenhagen, Copenhagen, Denmark; 18 Anthropology Department, University of Utah, Salt Lake City, UT USA; 19 Center for Computational Biology, University of California, Berkeley, CA USA; 20 Department of Geology, Lund University, Lund, Sweden; 21 Tårnby Gymnasium og HF, Kastrup, Denmark; 22 Department of Archaeology and Heritage Studies, Aarhus University, Aarhus, Denmark; 23 Department of Health Technology, Section of Bioinformatics, Technical University of Denmark, Kongens Lyngby, Denmark; 24 Vendsyssel Historiske Museum, Hjørring, Denmark; 25 Terra Ltd., Voronezh, Russian Federation; 26 Neurogenomics Division, The Translational Genomics Research Institute (TGEN), Phoenix, AZ USA; 27 Department of Archaeology, University of Exeter, Exeter, UK; 28 Institute of Archeology and Ethnography, Siberian Branch of the Russian Academy of Sciences, Novosibirsk, Russian Federation; 29 Department of Anthropology, Faculty of Biology, Lomonosov Moscow State University, Moscow, Russian Federation; 30 Department of Environmental Biology, Sapienza University of Rome, Rome, Italy; 31 Peter the Great Museum of Anthropology and Ethnography (Kunstkamera), Russian Academy of Sciences, Saint Petersburg, Russian Federation; 32 CIAS, Department of Life Sciences, University of Coimbra, Coimbra, Portugal; 33 UNIARQ, University of Lisbon, Lisbon, Portugal; 34 Kostanay Regional University A. Baitursynov, Kostanay, Kazakhstan; 35 Vesthimmerlands Museum, Aars, Denmark; 36 Grupo EvoAdapta, Departamento de Ciencias Históricas, Universidad de Cantabria, Santander, Spain; 37 Institute of Evolutionary Biology, CSIC-Universitat Pompeu Fabra, Barcelona, Spain; 38 Natural Sciences Museum of Barcelona (MCNB), Barcelona, Spain; 39 Departamento de Prehistoria y Arqueología, Universidad Autónoma de Madrid, Madrid, Spain; 40 Department of Biology, University of Rome Tor Vergata, Rome, Italy; 41 Instituto Internacional de Investigaciones Prehistóricas de Cantabria, Universidad de Cantabria, Banco Santander, Gobierno de Cantabria, Santander, Spain; 42 Centre de Recherche en Archéologie, Archeosciences, Histoire (CReAAH), UMR-6869 CNRS, Rennes, France; 43 Georgian National Museum, Tbilisi, Georgia; 44 Tbilisi State University, Tbilisi, Georgia; 45 IPND, Tyumen Scientific Centre, Siberian Branch of the Russian Academy of Sciences, Tyumen, Russian Federation; 46 Departament de Prehistòria, Arqueologia i Història Antiga, Universitat de València, València, Spain; 47 Department of Geological Sciences, University of Cape Town, Cape Town, South Africa; 48 Laboratory for Archaeological Chemistry, Department of Anthropology, University of Wisconsin–Madison, Madison, WI USA; 49 Department of Anthropology, New York University, New York, NY USA; 50 Institute of Humanities, Ivanovo State University, Ivanovo, Russian Federation; 51 Institute of Ethnology and Anthropology, Russian Academy of Sciences, Moscow, Russian Federation; 52 Saryarka Archaeological Institute, Buketov Karaganda University, Karaganda, Kazakhstan; 53 South Ural State University, Chelyabinsk, Russia; 54 A. Kh. Khalikov Institute of Archeology of the Academy of Sciences of the Republic of Tatarstan, Kazan, Russia; 55 Margulan Institute of Archaeology, Committee of Science of the Ministry of Science and Higher Education of the Republic of Kazakhstan, Almaty, Kazakhstan; 56 The Saxo Institute, University of Copenhagen, Copenhagen, Denmark; 57 Museum Østjylland, Randers, Denmark; 58 Soprintendenza Archeologia Belle Arti e Paesaggio per la Città Metropolitana di Bari, Bari, Italy; 59 UMR 5199 PACEA, CNRS, Université de Bordeaux, Pessac, France; 60 A.Kh. Margulan Institute of Archaeology, Almaty, Kazakhstan; 61 Institute of Archaeology, National Academy of Sciences of Ukraine, Kyiv, Ukraine; 62 National University of Kyiv-Mohyla Academy, Kyiv, Ukraine; 63 Collège de France, Paris, France; 64 Svendborg Museum, Svendborg, Denmark; 65 ICREA, University of Barcelona, Barcelona, Spain; 66 ARGEA Consultores SL, Madrid, Spain; 67 Museum Sydøstdanmark, Vordingborg, Denmark; 68 Institute of Archaeology and Ethnology, Polish Academy of Sciences, Kraków, Poland; 69 Department of Archaeology and Ancient History, Lund University, Lund, Sweden; 70 Aix-Marseille Université, CNRS, Min. Culture, UMR 7269, LAMPEA, Maison Méditerranéenne des Sciences de l’Homme, Aix-en-Provence, France; 71 Institute of Molecular Biology, National Academy of Sciences, Yerevan, Armenia; 72 Russian-Armenian University, Yerevan, Armenia; 73 HistorieUdvikler, Kalundborg, Denmark; 74 Department of History and Cultural Heritage, University of Siena, Siena, Italy; 75 Centre d’Anthropobiologie et de Génomique de Toulouse, CNRS UMR 5500, Université Paul Sabatier, Toulouse, France; 76 Soprintendenza per i Beni Archeologici delle Marche, Ancona, Italy; 77 Västergötlands Museum, Skara, Sweden; 78 Cabinet of Anthropology, Tomsk State University, Tomsk, Russian Federation; 79 Department of History, Humanities and Society, University of Rome Tor Vergata, Rome, Italy; 80 Faculty of Archaeology, Adam Mickiewicz University in Poznań, Poznań, Poland; 81 Institute of Archaeology, Jagiellonian University, Kraków, Poland; 82 Zoological Institute of Russian Academy of Sciences, Saint Petersburg, Russian Federation; 83 Department of Anthropology, Czech National Museum, Prague, Czech Republic; 84 Department of Health and Nature, University of Greenland, Nuuk, Greenland; 85 The Viking Ship Museum, Roskilde, Denmark; 86 Archaeology Institute, University of Highlands and Islands, Orkney, UK; 87 Scientific Research Center “Baikal region”, Irkutsk State University, Irkutsk, Russian Federation; 88 Laboratory for Experimental Traceology, Institute for the History of Material Culture of the Russian Academy of Sciences, Saint Petersburg, Russian Federation; 89 Janus Pannonius Museum, Pécs, Hungary; 90 Langelands Museum, Rudkøbing, Denmark; 91 Soprintendenza Archeologia, Belle Arti e Paesaggio per la provincia di Cosenza, Cosenza, Italy; 92 Paleoecology Laboratory, Institute of Plant and Animal Ecology, Ural Branch of the Russian Academy of Sciences, Ekaterinburg, Russian Federation; 93 Department of History of the Institute of Humanities, Ural Federal University, Ekaterinburg, Russian Federation; 94 Centre for the Study of Early Agricultural Societies, Department of Cross-Cultural and Regional Studies, University of Copenhagen, Copenhagen, Denmark; 95 Museum Nordsjælland, Hillerød, Denmark; 96 Museum Vestsjælland, Holbæk, Denmark; 97 Museum of Cultural History, University of Oslo, Oslo, Norway; 98 ArchaeoScience, Globe Institute, University of Copenhagen, Copenhagen, Denmark; 99 Department of History, University of Santiago de Compostela, Santiago de Compostela, Spain; 100 Lipetsk Regional Scientific Public Organisation “Archaeological Research”, Lipetsk, Russian Federation; 101 Institute of Archaeology and Ethnography, National Academy of Sciences, Yerevan, Armenia; 102 Center for Egyptological Studies, Russian Academy of Sciences, Moscow, Russian Federation; 103 Moesgaard Museum, Højbjerg, Denmark; 104 Nizhny Tagil State Socio-Pedagogical Institute, Nizhny Tagil, Russia; 105 Institute for History of Medicine, First Faculty of Medicine, Charles University, Prague, Czech Republic; 106 Centre for Archaeological Research, Toraighyrov University, Pavlodar, Kazakhstan; 107 Malmö Museer, Malmö, Sweden; 108 Institute of Statistical Sciences, School of Mathematics, University of Bristol, Bristol, UK; 109 Novo Nordisk Foundation Centre for Protein Research, Faculty of Health and Medical Sciences, University of Copenhagen, Copenhagen, Denmark; 110 Wellcome Sanger Institute, Hinxton, UK; 111 Department of Clinical Medicine, University of Copenhagen, Copenhagen, Denmark; 112 MARUM Center for Marine Environmental Sciences and Faculty of Geosciences, University of Bremen, Bremen, Germany

**Keywords:** Population genetics, Archaeology, Genomics

Correction to: *Nature* 10.1038/s41586-023-06865-0 Published online 10 January 2024

In the version of this article initially published, there were errors in the second affiliations for Levon Yepiskoposyan (Russian-Armenian University, Yerevan, Armenia) and Sergey Vasilyev (Center for Egyptological Studies, Russian Academy of Sciences, Moscow, Russian Federation), and in the first affiliation for Ruben Badalyan (Institute of Archaeology and Ethnography, National Academy of Sciences, Yerevan, Armenia); the affiliations are amended in the HTML and PDF versions of the article.

