# Supplementary material for: Population genomics of post-glacial western Eurasia
Source: Nature. 2024 Jan 10;625(7994):301–11. doi: 10.1038/s41586-023-06865-0 (PMC10781627; doi:10.1038/s41586-023-06865-0)
Supplement: Supplementary file 2 — Reporting Summary [file 41586_2023_6865_MOESM2_ESM.pdf]

nature portfolio

Corresponding author(s): Morten Allentoft, Martin Sikora, Eske Willerslev

Last updated by author(s): Sep 17, 2022

Reporting Summary

Nature Portfolio wishes to improve the reproducibility of the work that we publish. This form provides structure for consistency and transparency in reporting. For further information on Nature Portfolio policies, see our Editorial Policies and the Editorial Policy Checklist.

Statistics

For all statistical analyses, confirm that the following items are present in the figure legend, table legend, main text, or Methods section.

n/a	Confirmed	
☐	☒	The exact sample size (n) for each experimental group/condition, given as a discrete number and unit of measurement
☐	☒	A statement on whether measurements were taken from distinct samples or whether the same sample was measured repeatedly
☐	☒	The statistical test(s) used AND whether they are one- or two-sided<br>*Only common tests should be described solely by name; describe more complex techniques in the Methods section.*
☐	☒	A description of all covariates tested
☐	☒	A description of any assumptions or corrections, such as tests of normality and adjustment for multiple comparisons
☐	☒	A full description of the statistical parameters including central tendency (e.g. means) or other basic estimates (e.g. regression coefficient) AND variation (e.g. standard deviation) or associated estimates of uncertainty (e.g. confidence intervals)
☐	☒	For null hypothesis testing, the test statistic (e.g. F, t, r) with confidence intervals, effect sizes, degrees of freedom and P value noted<br>*Give P values as exact values whenever suitable.*
☐	☒	For Bayesian analysis, information on the choice of priors and Markov chain Monte Carlo settings
☐	☒	For hierarchical and complex designs, identification of the appropriate level for tests and full reporting of outcomes
☐	☒	Estimates of effect sizes (e.g. Cohen's d, Pearson's r), indicating how they were calculated

Our web collection on statistics for biologists contains articles on many of the points above.

Software and code

Policy information about availability of computer code

Data collection

Sequencing data and metadata pertaining to the sequencing of ancient genomes is managed on secure servers at the Globe Institute, University of Copenhagen, and via the Illumina Inc. BaseSpace platform. Accelerator Mass Spectrometry data and associated metadata is managed at the Department of Historical Studies, University of Gothenberg.

Data analysis

Custom scripts used to apply chromopainter from large-scale phased data are available at https://github.com/will-camb/Nero/tree/master/scripts/cp_panel_scripts. All other analyses relied upon available software which has been fully referenced in the manuscript and detailed in the relevant supplementary notes. These comprise:
CASAVA (v.1.8.2)
AdapterRemoval (v.2.1.3)
Picard (v.1.127)
GATK (v.3.3.0)
Samtools (1.9)
Samtools calmd (v.1.10)
pysam (https://github.com/pysam-developers/pysam)
BEDtools (v.2.23.0)
mapDamage2.0 (v2.2.1)
BWA (0.7-17)
Schmutzi (VERSION?)
ContamMix (VERSION?)
ANGSD (0.938)
GLIMPSE (v1.0.1)

Beagle (v4.1)
BCFtools 1.10
MAFFT (v7.490)
RAxML-ng (v1.1.0)
ry_compute (v0.4)
EPA-ng (0.3.8)
NgsRelate (v2)
ADMIXTURE (1.3.0)
smartpca
GCTA
R version 4.0
ADMIXTOOLS2
gstat (2.0-9)
IBDseq (version r1206)
GenomicRanges (3.15)
leidenAlg (v1.01, https://github.com/kharchenkolab/leidenAlg)
igraph (0.9.9)
limSolve (1.5.6)
Scikit-learn (0.21.2)
Chromopainter (0.0.4)
fineSTRUCTURE (0.0.5)
QCTOOL v2 (https://www.well.ox.ac.uk/~gav/qctool_v2/)
ArcGIS Online (www.arcgis.com)
OxCal v4.4
LRA v0.1.0 (https://github.com/petrkunes/LRA)

For manuscripts utilizing custom algorithms or software that are central to the research but not yet described in published literature, software must be made available to editors and reviewers. We strongly encourage code deposition in a community repository (e.g. GitHub). See the Nature Portfolio guidelines for submitting code & software for further information.

Data

Policy information about availability of data

All manuscripts must include a data availability statement. This statement should provide the following information, where applicable:

- Accession codes, unique identifiers, or web links for publicly available datasets
- A description of any restrictions on data availability
- For clinical datasets or third party data, please ensure that the statement adheres to our policy

All collapsed and paired-end sequence data for novel samples sequenced in this study will be made publicly available on the European Nucleotide Archive, together with trimmed sequence alignment map files, aligned using human build GRCh37. Previously published ancient genomic data used in this study is detailed in Supplementary Table VII, and are all already publicly available. Bioarchaeological data (including stable isotopes and radiocarbon dating results) are included in the online supplementary materials of this submission.

Human research participants

Policy information about studies involving human research participants and Sex and Gender in Research.

Reporting on sex and gender	No living or recently deceased human research participants were affected by this study
Population characteristics	*Describe the covariate-relevant population characteristics of the human research participants (e.g. age, genotypic information, past and current diagnosis and treatment categories). If you filled out the behavioural & social sciences study design questions and have nothing to add here, write "See above."*
Recruitment	*Describe how participants were recruited. Outline any potential self-selection bias or other biases that may be present and how these are likely to impact results.*
Ethics oversight	*Identify the organization(s) that approved the study protocol.*

Note that full information on the approval of the study protocol must also be provided in the manuscript.

Field-specific reporting

Please select the one below that is the best fit for your research. If you are not sure, read the appropriate sections before making your selection.

☐ Life sciences ☐ Behavioural & social sciences ☒ Ecological, evolutionary & environmental sciences

For a reference copy of the document with all sections, see nature.com/documents/nr-reporting-summary-flat.pdf

Ecological, evolutionary & environmental sciences study design

All studies must disclose on these points even when the disclosure is negative.

Study description	We sequenced 317 novel ancient human genomes from primarily Mesolithic and Neolithic individuals across Eurasia, combined with new radiocarbon dates, stable isotope data. We additionally test and apply GLIMPSE to impute 1492 previously published ancient genomes, and incorporate this data in our analyses. We undertake a suite of analyses of this data to generate new insights into population structure through time, natural selection and health-related adaptation.
Research sample	Ancient human remains (bone and dental cementum) were collected, sampled, extracted and screened by shallow shotgun sequencing (see Supplementary Note 1). This resulted in 317 samples found to be suitable for deep sequencing (of 962 initial specimens). These represent a considerable geographic range across Eurasia (from Lake Baikal to the Atlantic coast). A high sample density for Denmark (100) was undertaken to provide high resolution insights at this location. A particular focus upon individuals related to Mesolithic or Neolithic culture was made, with 300 of the individuals from the time span 11,000 cal. BP to 3,000 cal. BP.
Sampling strategy	Sampling was dependent upon the availability of ancient human remains, though a considerable transect of the Mesolithic and Neolithic periods in Eurasia was represented, together with a detailed continuous sequence of human occupation of Denmark specifically. Our assemblage is well-represented with individuals of such key archaeological complexes as the Maglemose, Ertebølle and Funnel Beaker cultures in Scandinavia, the Cardial in the Mediterranean, the Körös and Linear Pottery complexes in SE and Central Europe, and many archaeological cultures in Ukraine, western Russia, and the trans-Ural (e.g. Veretye, Lyalovo, Volosovo, Kitoi).
Data collection	Sampled remains consisted of teeth (sampled for dental cementum, N=211), petrous bones (N=91), and other bone types (N=15; long bones, ribs and cranial bones). Sampling and ancient DNA laboratory work was undertaken in dedicated ancient DNA clean lab facilities at the GeoGenetics Centre. Additionally, 272 novel accelerator mass spectrometry results were generated at the 14CHRONO laboratory, Queen's University Belfast (242 samples), at the Oxford Radiocarbon Accelerator Unit (ORAU) laboratory (24 samples) and at the Keck-CCAMS Group, Irvine, California, USA (6 samples).
Timing and spatial scale	Sample chronology was generated by radiocarbon dating, with dates corrected for marine and freshwater reservoir effects. These showed samples ranging from the Upper Palaeolithic (c. 25,700 cal. BP) to the mediaeval period (c. 1200 cal. BP). Most individuals (97%, N=309) span 11,000 cal. BP to 3,000 cal. BP, the period broadly associated with the Mesolithic and Neolithic in Eurasia. Our research area can broadly be divided into three large regions: 1) central, western and northern Europe, 2) eastern Europe including western Russia and Ukraine, and 3) the Urals and western Siberia.
Data exclusions	Samples that were screened by shallow sequencing and found to have endogenous DNA of >1% were not sequenced further and not included in analyses. This amounted to 645 specimens.
Reproducibility	Data quality and uncertainty (e.g. contamination) was accounted for in computational analyses to assess robustness of inferences, and all methods and data are made available for future replication.
Randomization	Sample groups were defined to reflect archaeological populations, based on phylogenetic inferences, temporal and geographic provenance, and cultural interpretations evidenced by archaeological contexts.
Blinding	Blinding was not applicable to this study.

Did the study involve field work? ☐ Yes ☒ No

Reporting for specific materials, systems and methods

We require information from authors about some types of materials, experimental systems and methods used in many studies. Here, indicate whether each material, system or method listed is relevant to your study. If you are not sure if a list item applies to your research, read the appropriate section before selecting a response.

Materials & experimental systems

n/a	Involved in the study	
☒	☐	Antibodies
☒	☐	Eukaryotic cell lines
☐	☒	Palaeontology and archaeology
☒	☐	Animals and other organisms
☒	☐	Clinical data
☒	☐	Dual use research of concern

Methods

n/a	Involved in the study	
☒	☐	ChIP-seq
☒	☐	Flow cytometry
☒	☐	MRI-based neuroimaging

Palaeontology and Archaeology

Specimen provenance

Samples analysed in this study represent a considerable transect of Eurasian Mesolithic and Neolithic archaeological sites. Institution: 176 in total. Given the highly collaborative, international scale of the sampling effort, key contact persons for each site helped facilitate the access to material and coordinate legal authorisation for accessing sample material. Samples were collected with permission from organisations holding specimens and documented via permissions or agreements letters held by the Lundbeck Foundation GeoGenetics Centre. Full details and documentation for these is maintained in a dedicated database by F.B., K.G.S and Pernille Olsen of the GeoGenetics Centre. The following list provides the site name, country, sample ID, contact and institution. Further details of each site and excavation history are provided in Supplementary Notes 5-6.

Afontova Gora, Russia. Samples: NEO102. Key contact: Mikhail Sablin. Institution: Zoological Institute of the Russian Academy of Sciences.
Aknashen, Armenia. Samples: NEO110. Key contact: Levon Yeppiskoposyan. Institution: National Academy of Sciences of Armenia, Laboratory of Ethnogenomics, Yerevan, Armenia.
Ängdala, Sweden. Samples: NEO046. Key contact: Catharina Ödman / Yvonne Magnusson. Institution: Malmö museum.
Avlebjerg, Denmark. Samples: NEO961. Key contact: Anders Fischer. Institution: Sealand Archaeology/University of Copenhagen.
Baile herculane, Romania. Samples: NEO674. Key contact: Dušan Borić. Institution: Columbia university.
Banks Tomb, UK. Samples: NEO624, NEO625, NEO626, NEO627, NEO630, NEO717. Key contact: Nick Card. Institution: Orkney Research Centre for Archaeology. Institution: ORCA.
Bazaiha, Russia. Samples: NEO070. Key contact: Mikhail Sablin. Institution: Zoological Institute of the Russian Academy of Sciences.
Biestamak, Kazakhstan. Samples: NEO057. Key contact: Emma Usmanova, Irina Shevnina, Andrey Logvin. Institution: Karaganda State University.
Bjørnsholm, Denmark. Samples: NEO751. Key contact: Niels Lynnerup, Anders Fischer, Pia Bennike. Institution: Sealand Archaeology/ University of Copenhagen.
Bodal K, Denmark. Samples: NEO814. Key contact: Lasse Sørensen, Peter Vang, Anders Fischer. Institution: National Museum of Denmark
Bol'shoy Oleni Ostrov, Russia. Samples: NEO060, NEO061, NEO062. Key contact: Vyacheslav Moiseyev. Institution: Peter the Great Museum of Anthropology and ethnography (Kunstkamera).
Borovjanka XVII, Russia. Samples: NEO080, NEO081, NEO083. Key contact: Vyacheslav Moiseyev. Institution: Peter the Great Museum of Anthropology and Ethnography (Kunstkamera).
Borreby, Denmark. Samples: NEO735, NEO737. Key contact: Niels Lynnerup, Anders Fischer, Pia Bennike. Institution: Sealand Archaeology/University of Copenhagen.
Bredgården, Sweden. Samples: NEO027. Key contact: Maria Vretemark. Institution: Västergötlands museum.
Bybjerg, Denmark. Samples: NEO563. Key contact: Niels Lynnerup, Anders Fischer, Pia Bennike. Institution: Sealand Archaeology/ University of Copenhagen.
Bygholm, Denmark. Samples: NEO564. Key contact: Niels Lynnerup, Anders Fischer, Pia Bennike. Institution: Sealand Archaeology/ University of Copenhagen.
Camino de las Yeseras, Spain. Samples: NEO721. Key contact: Corina Liesau, Pilar Prieto. Institution: Universidad Autónoma de Madrid.
Dalmosegaard, Denmark. Samples: NEO886. Key contact: Niels Lynnerup, Anders Fischer, Pia Bennike. Institution: Sealand Archaeology/University of Copenhagen.
Dezsk, Hungary. Samples: NEO130. Key contact: Vaclav Smrcka. Institution: Institute for History of Medicine and Foreign Languages of the First Faculty of Medicine, Charles University.
Døjringe, Denmark. Samples: NEO566. Key contact: Niels Lynnerup, Anders Fischer, Pia Bennike. Institution: Sealand Archaeology/ University of Copenhagen.
Dolgoe Ozero, Russia. Samples: NEO292. Key contact: Sergey Vasilyev. Institution: Inst. of Ethnography and Anthropology, Moscow.
Dösemarken, Sweden. Samples: NEO044. Key contact: Catharina Ödman / Yvonne Magnusson. Institution: Malmö museum.
Dragsholm, Denmark. Samples: NEO822. Key contact: Niels Lynnerup, Anders Fischer, Pia Bennike. Institution: Danish National Museum.
Dragsholm, Denmark. Samples: NEO732, NEO733. Key contact: Niels Lynnerup, Anders Fischer, Pia Bennike. Institution: Sealand Archaeology/University of Copenhagen.
El Mazo, Spain. Samples: NEO646, NEO938. Key contact: Igor Gutiérrez Zugasti. Institution: Universidad de Cantabria.
Elkenøre, Denmark. Samples: NEO888. Key contact: Niels Lynnerup, Anders Fischer, Pia Bennike. Institution: Sealand Archaeology/ University of Copenhagen.
Ertebølle, Denmark. Samples: NEO568, NEO569. Key contact: Niels Lynnerup, Anders Fischer, Pia Bennike. Institution: Sealand Archaeology/University of Copenhagen.
Evensås, Sweden. Samples: NEO260. Key contact: Karl-Göran Sjögren. Institution: Gothenburg university.
Falköping 5, Sweden. Samples: NEO220, NEO221, NEO223, NEO224, NEO225, NEO226, NEO227, NEO228. Key contact: Malou Blank. Institution: Gothenburg university.
Fannerup D, Denmark. Samples: NEO855. Key contact: Lutz Klassen. Institution: Museum Østjylland.
Fannerup E, Denmark. Samples: NEO570. Key contact: Niels Lynnerup, Anders Fischer, Pia Bennike. Institution: Sealand Archaeology/ University of Copenhagen.
Fannerup F, Denmark. Samples: NEO930. Key contact: Lutz Klassen / Rikke Maring. Institution: Museum Østjylland.
Femhøve Vig, Denmark. Samples: NEO744. Key contact: Niels Lynnerup, Anders Fischer, Pia Bennike. Institution: Sealand Archaeology/University of Copenhagen.
Fofonovo, Russia. Samples: NEO199, NEO200, NEO201. Key contact: Sergey Vasilyev. Institution: Inst. of Ethnography and Anthropology, Moscow.
Fontenoce, Italy. Samples: NEO830. Key contact: Gabriele Scorrano. Institution: University of Copenhagen.
Frälsegården, Sweden. Samples: NEO259. Key contact: Karl-Göran Sjögren. Institution: Gothenburg university.
Gaudo, Italy. Samples: NEO828. Key contact: Gabriele Scorrano. Institution: University of Copenhagen.
Gazel Cave, France. Samples: NEO812, NEO813. Key contact: Patrice Cortaud. Institution: Universite de Bordeaux.
Golubaya Krinitsa, Russia. Samples: NEO204, NEO207, NEO209, NEO210, NEO212. Key contact: Ruslan Turin. Institution: Karaganda

State University.
Golubaya Krinitsa, Russia. Samples: NEO113. Key contact: Sergey Vasilyev. Institution: Inst. of Ethnography and Anthropology, Moscow.
Gorzsa Cukormajor, Hungary. Samples: NEO140, NEO142, NEO143, NEO145, NEO147, NEO148, NEO149. Key contact: Vaclav Smrcka. Institution: Institute for History of Medicine and Foreign Languages of the First Faculty of Medicine, Charles University.
Gregorievka 1, Kazakhstan. Samples: NEO899. Key contact: Victor Merc/Emma Usmanova. Institution: Karaganda State University.
Grøfte, Denmark. Samples: NEO571. Key contact: Niels Lynnerup, Anders Fischer, Pia Bennike. Institution: Sealand Archaeology/ University of Copenhagen.
Grotta Delle Mura, Italy. Samples: NEO806. Key contact: Gabriele Scorrano. Institution: University of Copenhagen.
Grotta Nisco, Italy. Samples: NEO823. Key contact: Gabriele Scorrano. Institution: University of Copenhagen.
Gruta do Caldeirão, Portugal. Samples: NEO631, NEO632. Key contact: Joao Zilhao. Institution: Universitat de Barcelona.
Hanaskede, Sweden. Samples: NEO018. Key contact: Maria Vretemark. Institution: Västergötlands museum.
Havnø, Denmark. Samples: NEO941. Key contact: Anders Fischer/Niels Lynnerup. Institution: Sealand Archaeology/University of Copenhagen.
Hedegaard. Institution: Bislev), Denmark. Samples: NEO013. Key contact: Bjarne Nielsen.
Henriksholm-Bøgebakken. Institution: Vedbæk), Denmark. Samples: NEO745, NEO746, NEO747, NEO748, NEO749. Key contact: Niels Lynnerup, Anders Fischer, Pia Bennike. Institution: Sealand Archaeology/University of Copenhagen.
Hetty Pegler's Tump, UK. Samples: NEO847. Key contact: Alan Outram. Institution: University of Exeter.
Hindby Mosse, Sweden. Samples: NEO036, NEO038, NEO039. Key contact: Catharina Ödman / Yvonne Magnusson. Institution: Malmö museum.
Hödmezövàsàhely Kotac, Hungary. Samples: NEO137. Key contact: Vaclav Smrcka. Institution: Institute for History of Medicine and Foreign Languages of the First Faculty of Medicine, Charles University.
Holmegård-Djursland, Denmark. Samples: NEO001. Key contact: Anders Fischer, Pia Bennike. Institution: Sealand Archaeology/ University of Copenhagen.
Hove Å, Denmark. Samples: NEO946. Key contact: Anders Fischer/Niels Lynnerup. Institution: Sealand Archaeology/University of Copenhagen.
Hummervik, Norway. Samples: NEO017. Key contact: Per Åke Persson. Institution: UiO: Kulturhistorisk museum.
Igren, Ukraine. Samples: NEO509, NEO516, NEO518, NEO521. Key contact: Alexandra Buzhilova. Institution: Lomonosov Moscow State University.
Itkul', Russia. Samples: NEO063, NEO064, NEO065. Key contact: Vyacheslav Moiseyev. Institution: Peter the Great Museum of Anthropology and Ethnography (Kunstkamera).
Jorløse Mose, Denmark. Samples: NEO023. Key contact: Anders Fischer, Pia Bennike. Institution: Sealand Archaeology/University of Copenhagen.
Jørlundegaard, Denmark. Samples: NEO702. Key contact: Niels Lynnerup, Anders Fischer, Pia Bennike. Institution: Sealand Archaeology/University of Copenhagen.
Kainsbakke, Denmark. Samples: NEO025. Key contact: Lutz Klassen. Institution: Museum Østjylland.
Karavaikha, Russia. Samples: NEO555, NEO556, NEO557, NEO558, NEO559, NEO560, NEO561. Key contact: Alexandra Buzhilova. Institution: Lomonosov Moscow State University.
Kastanjegården, Sweden. Samples: NEO051. Key contact: Catharina Ödman / Yvonne Magnusson. Institution: Malmö museum.
Klæstrup Holm, Denmark. Samples: NEO951. Key contact: Anders Fischer/Niels Lynnerup. Institution: Sealand Archaeology/University of Copenhagen.
Kleshnya III 1998, Ukraine. Samples: NEO278. Key contact: Inna Potekhina. Institution: Institute of Archaeology of Ukrainian National Academy of Sciences.
Klokkehøj, Denmark. Samples: NEO580. Key contact: Niels Lynnerup, Anders Fischer, Pia Bennike. Institution: Sealand Archaeology/ University of Copenhagen.
Koed I, Denmark. Samples: NEO583. Key contact: Niels Lynnerup, Anders Fischer, Pia Bennike. Institution: Sealand Archaeology/ University of Copenhagen.
Koed IV, Denmark. Samples: NEO586. Key contact: Niels Lynnerup, Anders Fischer, Pia Bennike. Institution: Sealand Archaeology/ University of Copenhagen.
Koelbjerg, Denmark. Samples: NEO254. Key contact: Jesper Hansen. Institution: Odense Bys Museer.
Køge Sønakke, Denmark. Samples: NEO759. Key contact: Niels Lynnerup, Anders Fischer, Pia Bennike. Institution: Sealand Archaeology/University of Copenhagen.
Kolind, Denmark. Samples: NEO738, NEO739. Key contact: Niels Lynnerup, Anders Fischer, Pia Bennike. Institution: Sealand Archaeology/University of Copenhagen.
Kongemose, Denmark. Samples: NEO587. Key contact: Niels Lynnerup, Anders Fischer, Pia Bennike. Institution: Sealand Archaeology/ University of Copenhagen.
Korsør Glasværk, Denmark. Samples: NEO589. Key contact: Niels Lynnerup, Anders Fischer, Pia Bennike. Institution: Sealand Archaeology/University of Copenhagen.
Korsør Nor, Denmark. Samples: NEO791. Key contact: Lasse Sørensen, Poul Otto Nielsen. Institution: National Museum of Denmark.
Kotias Klde Cave, Georgia. Samples: NEO281, NEO283. Key contact: David O. Lordkipanidze. Institution: Georgian National Museum.
Ksizovo 6, Russia. Samples: NEO172, NEO173, NEO174, NEO175. Key contact: Sergey Vasilyev. Institution: Inst. of Ethnography and Anthropology, Moscow.
Kumyshanskaya Cave, Russia. Samples: NEO687. Key contact: Andrej Evteev. Institution: Moscow State University.
Kyndeløse, Denmark. Samples: NEO878. Key contact: Niels Lynnerup, Anders Fischer, Pia Bennike. Institution: Sealand Archaeology/ University of Copenhagen.
Læsten Mose, Denmark. Samples: NEO945. Key contact: Anders Fischer/Niels Lynnerup. Institution: Sealand Archaeology/University of Copenhagen.
Langø Skaldynge, Denmark. Samples: NEO853. Key contact: Anders Fischer/ Pia Bennike. Institution: Sealand Archaeology/University of Copenhagen.
Lendemark, Denmark. Samples: NEO896. Key contact: Niels Lynnerup, Anders Fischer, Pia Bennike. Institution: Sealand Archaeology/ University of Copenhagen.
Lepenski Vir, Serbia. Samples: NEO669. Key contact: Dušan Borić. Institution: Columbia university.
Lohals Nord, Denmark. Samples: NEO029. Key contact: Anders Fischer, Pia Bennike. Institution: Sealand Archaeology/University of Copenhagen.
Lollikehuse, Denmark. Samples: NEO857. Key contact: Kristian Gregersen. Institution: Statens Naturhistoriske Museum.
Lundby-Falster, Denmark. Samples: NEO865, NEO866. Key contact: Niels Lynnerup, Anders Fischer, Pia Bennike. Institution: Sealand

Archaeology/University of Copenhagen.
Lysa Gora, Ukraine. Samples: NEO262, NEO265. Key contact: Inna Potekhina. Institution: Institute of Archaeology of Ukrainian National Academy of Sciences.
Maddalena, Italy. Samples: NEO695. Key contact: Alfredo Coppa. Institution: Universita di Roma Sapienza.
Madesø, Denmark. Samples: NEO752. Key contact: Niels Lynnerup, Anders Fischer, Pia Bennike. Institution: Sealand Archaeology/ University of Copenhagen.
Magleø, Denmark. Samples: NEO590. Key contact: Niels Lynnerup, Anders Fischer, Pia Bennike. Institution: Sealand Archaeology/ University of Copenhagen.
Mamaj Gora, Ukraine. Samples: NEO268, NEO270. Key contact: Inna Potekhina. Institution: Institute of Archaeology of Ukrainian National Academy of Sciences.
Mandrin Cave, France. Samples: NEO119, NEO120, NEO121. Key contact: Ludovic Slimak. Institution: Université Toulouse.
Mandzuli-Depe, Turkmenistan. Samples: NEO310. Key contact: Sergey Vasilyev. Institution: Inst. of Ethnography and Anthropology, Moscow.
Mergen' 6, Russia. Samples: NEO072, NEO073. Key contact: Vyacheslav Moiseyev. Institution: Peter the Great Museum of Anthropology and ethnography (Kunstkamera).
Minino, Russia. Samples: NEO536, NEO537, NEO538, NEO539. Key contact: Alexandra Buzhilova. Institution: Lomonosov Moscow State University.
Mora Cavorso, Italy. Samples: NEO834. Key contact: Gabriele Scorrano. Institution: University of Copenhagen.
Mosede Mose. Institution: Karlslunde Mose), Denmark. Samples: NEO860. Key contact: Niels Lynnerup, Anders Fischer, Pia Bennike. Institution: Sealand Archaeology/University of Copenhagen.
Mosede Mose III, Denmark. Samples: NEO861. Key contact: Niels Lynnerup, Anders Fischer, Pia Bennike. Institution: Sealand Archaeology/University of Copenhagen.
Myrebjerg Mose, Denmark. Samples: NEO925. Key contact: Otto Christian Uldum. Institution: Langelands Museum.
Næs, Denmark. Samples: NEO792. Key contact: Lasse Sørensen, Poul Otto Nielsen. Institution: National Museum of Denmark.
Nederst, Denmark. Samples: NEO856. Key contact: Lutz Klassen. Institution: Museum Østjylland.
Neverkær Mose I, Denmark. Samples: NEO594. Key contact: Niels Lynnerup, Anders Fischer, Pia Bennike. Institution: Sealand Archaeology/University of Copenhagen.
Norsminde, Denmark. Samples: NEO852. Key contact: Anders Fischer/ Pia Bennike. Institution: Sealand Archaeology/University of Copenhagen.
Okunevo 5, Russia. Samples: NEO068, NEO077. Key contact: Vyacheslav Moiseyev. Institution: Peter the Great Museum of Anthropology and ethnography (Kunstkamera).
Okunevo 7, Russia. Samples: NEO079. Key contact: Vyacheslav Moiseyev. Institution: Peter the Great Museum of Anthropology and ethnography (Kunstkamera).
Omskaya Stoyanka II, Russia. Samples: NEO075, NEO078. Key contact: Vyacheslav Moiseyev. Institution: Peter the Great Museum of Anthropology and ethnography (Kunstkamera).
Orehoved Sejlrende, Denmark. Samples: NEO122, NEO123. Key contact: Anders Fischer, Pia Bennike, Morten Johansen. Institution: Sealand Archaeology/University of Copenhagen.
Ostrov 2, Russia. Samples: NEO076. Key contact: Vyacheslav Moiseyev. Institution: Peter the Great Museum of Anthropology and ethnography (Kunstkamera).
Pad Tokui, Russia. Samples: NEO116. Key contact: Sergey Vasilyev. Institution: Inst. of Ethnography and Anthropology, Moscow.
Pandebjerg, Denmark. Samples: NEO595. Key contact: Niels Lynnerup, Anders Fischer, Pia Bennike. Institution: Sealand Archaeology/ University of Copenhagen.
Peschanitsa, Russia. Samples: NEO202. Key contact: Sergey Vasilyev. Institution: Inst. of Ethnography and Anthropology, Moscow.
Pogostishche, Russia. Samples: NEO554. Key contact: Alexandra Buzhilova. Institution: Lomonosov Moscow State University.
Porsmose, Denmark. Samples: NEO795. Key contact: Lasse Sørensen, Poul Otto Nielsen. Institution: National Museum of Denmark.
Protoka, Russia. Samples: NEO309. Key contact: Sergey Vasilyev. Institution: Inst. of Ethnography and Anthropology, Moscow.
Ravnsbjerggård, Denmark. Samples: NEO960. Key contact: Anders Fischer. Institution: Sealand Archaeology/University of Copenhagen.
Rødhals (Sejerø), Denmark. Samples: NEO645. Key contact: Niels Lynnerup, Anders Fischer, Pia Bennike. Institution: Sealand Archaeology/University of Copenhagen.
Røntesten, Denmark. Samples: NEO019. Key contact: Anders Fischer, Pia Bennike. Institution: Sealand Archaeology/University of Copenhagen.
Roskilde Fjord, Denmark. Samples: NEO891. Key contact: Niels Lynnerup, Anders Fischer, Pia Bennike. Institution: Sealand Archaeology/University of Copenhagen.
Rude, Denmark. Samples: NEO041, NEO043. Key contact: Niels Lynnerup. Institution: Department of Forensic Medicine, Copenhagen university.
Sakhtish 8, Russia. Samples: NEO184. Key contact: Sergey Vasilyev. Institution: Inst. of Ethnography and Anthropology, Moscow.
Sakhtish II, Russia. Samples: NEO158, NEO178, NEO179, NEO192. Key contact: Sergey Vasilyev. Institution: Inst. of Ethnography and Anthropology, Moscow.
Sakhtish IIa, Russia. Samples: NEO180, NEO181, NEO182, NEO183, NEO185, NEO186, NEO187, NEO188, NEO189, NEO193, NEO194, NEO195, NEO197. Key contact: Sergey Vasilyev. Institution: Inst. of Ethnography and Anthropology, Moscow.
Salpetermosen, Denmark. Samples: NEO028. Key contact: Niels Lynnerup/Thomas Jørgensen.
Santa Maira, Spain. Samples: NEO694. Key contact: Carles Lalueza-Fox. Institution: Pompeu Fabra.
Sao Paulo II, Portugal. Samples: NEO603, NEO609. Key contact: Ana Maria Silva. Institution: University of Coimbra.
Schela Cladovei, Romania. Samples: NEO671, NEO672. Key contact: Dušan Borić. Institution: Columbia university.
Sejerby (Sejerø), Denmark. Samples: NEO757. Key contact: Niels Lynnerup, Anders Fischer, Pia Bennike. Institution: Sealand Archaeology/University of Copenhagen.
Sigersdal, Denmark. Samples: NEO007. Key contact: Anders Fischer, Pia Bennike. Institution: Sealand Archaeology/University of Copenhagen.
Sigersdal Mose, Denmark. Samples: NEO753. Key contact: Niels Lynnerup, Anders Fischer, Pia Bennike. Institution: Sealand Archaeology/University of Copenhagen.
Sillvik, Sweden. Samples: NEO261. Key contact: Karl-Göran Sjögren. Institution: Gothenburg university.
Sjauke, Kazakhstan. Samples: NEO900. Key contact: Victor Merc/Emma Usmanova. Institution: Karaganda State University.
Sjauke 1, Kazakhstan. Samples: NEO902. Key contact: Victor Merc/Emma Usmanova. Institution: Karaganda State University.
Sjauke settlement, Kazakhstan. Samples: NEO901. Key contact: Victor Merc/Emma Usmanova. Institution: Karaganda State University.

Sjiderti 10, Kazakhstan. Samples: NEO904. Key contact: Victor Merc/Emma Usmanova. Institution: Karaganda State University.
Skateholm I, Sweden. Samples: NEO679. Key contact: Douglas Price / Lars Larsson,. Institution: Lund university.
Słonowice, Poland. Samples: NEO640, NEO641. Key contact: Piotr Wlodarczak. Institution: Polish Academy of Sciences. Institution: Instytut Archeologii i Etnologii.
Sludegård Sømose, Denmark. Samples: NEO933. Key contact: Anders Fischer / Jesper Hansen. Institution: Odense Bys museer.
Sølager, Denmark. Samples: NEO598. Key contact: Niels Lynnerup, Anders Fischer, Pia Bennike. Institution: Sealand Archaeology/University of Copenhagen.
Sope-2, Estonia. Samples: NEO306. Key contact: Sergey Vasilyev. Institution: Inst. of Ethnography and Anthropology, Moscow.
Sosnovyiy Myis, Russia. Samples: NEO841, NEO843. Key contact: Vyacheslav Moiseyev. Institution: Peter the Great Museum of Anthropology and ethnography (Kunstkamera).
Stenderup Hage, Denmark. Samples: NEO943. Key contact: Anders Fischer/Niels Lynnerup.
Storelyng. Institution: Øgårde boat III), Denmark. Samples: NEO597. Key contact: Niels Lynnerup, Anders Fischer, Pia Bennike. Institution: Sealand Archaeology/University of Copenhagen.
Storelyng (Østrup Homo II), Denmark. Samples: NEO602. Key contact: Niels Lynnerup, Anders Fischer, Pia Bennike. Institution: Sealand Archaeology/University of Copenhagen.
Strøby Egede, Denmark. Samples: NEO092. Key contact: Niels Lynnerup. Institution: Department of Forensic Medicine, Copenhagen university.
Strøby Grøftemark, Denmark. Samples: NEO091. Key contact: Niels Lynnerup, Anders Fischer. Institution: Department of Forensic Medicine, Copenhagen university.
Strøby Ladeplads, Denmark. Samples: NEO093. Key contact: Niels Lynnerup. Institution: Department of Forensic Medicine, Copenhagen university.
Svinninge Vejle, Denmark. Samples: NEO898. Key contact: Niels Lynnerup, Anders Fischer, Pia Bennike. Institution: Sealand Archaeology/University of Copenhagen.
Tepe Guran, Iran. Samples: NEO816, NEO817, NEO819. Key contact: Peder Mortensen, Pernille Bangsgaard. Institution: Statens Naturhistoriske Museum.
Tingbjerggård Vest, Denmark. Samples: NEO957. Key contact: Anders Fischer/Niels Lynnerup. Institution: Sealand Archaeology/University of Copenhagen.
Tissøe, Denmark. Samples: NEO942. Key contact: Anders Fischer/Niels Lynnerup. Institution: Sealand Archaeology/University of Copenhagen.
Toftum, Skanderborg, Denmark. Samples: NEO870, NEO872, NEO875, NEO876. Key contact: Niels Lynnerup, Anders Fischer, Pia Bennike. Institution: Sealand Archaeology/University of Copenhagen.
Toral III, Spain. Samples: NEO648, NEO649, NEO650, NEO653. Key contact: Igor Gutiérrez Zugasti. Institution: Universidad de Cantabria.
Troldebjerg, Denmark. Samples: NEO934. Key contact: Anders Fischer/Otto Uldum. Institution: Langelands Museum, Rudkøbing.
Tudse Hage, Denmark. Samples: NEO932. Key contact: Anders Fischer. Institution: Museum Vestsjælland, Museum Vestsjælland.
Tybrind Vig, Denmark. Samples: NEO683. Key contact: Anders Fischer. Institution: Sealand Archaeology/University of Copenhagen.
Tysmosen II, Denmark. Samples: NEO790. Key contact: Lasse Sørensen, Anders Fischer, Poul Otto Nielsen. Institution: National Museum of Denmark.
Ural River Beach, Russia. Samples: NEO100. Key contact: Mikhail Sablin. Institution: Zoological Institute of the Russian Academy of Sciences.
Ust'-Isha, Russia. Samples: NEO067. Key contact: Vyacheslav Moiseyev. Institution: Peter the Great Museum of Anthropology and ethnography (Kunstkamera).
Vængesø II, Denmark. Samples: NEO003. Key contact: Anders Fischer, Pia Bennike. Institution: Sealand Archaeology/University of Copenhagen.
Vanløse Mose II, Denmark. Samples: NEO599. Key contact: Niels Lynnerup, Anders Fischer, Pia Bennike. Institution: Sealand Archaeology/University of Copenhagen.
Vasagård, Denmark. Samples: NEO815. Key contact: Lasse Sørensen. Institution: National Museum of Denmark.
Vasilevka-I, Ukraine. Samples: NEO492, NEO494, NEO496, NEO497, NEO501, NEO545, NEO549, NEO550. Key contact: Alexandra Buzhilova. Institution: Lomonosov Moscow State University.
Vasilevsky, Ukraine. Samples: NEO305. Key contact: Sergey Vasilyev. Institution: Inst. of Ethnography and Anthropology, Moscow.
Vasilyevskiy kordon 17, Russia. Samples: NEO160, NEO162, NEO163, NEO164, NEO166, NEO167, NEO168, NEO170, NEO171. Key contact: Sergey Vasilyev. Institution: Inst. of Ethnography and Anthropology, Moscow.
Vattenledningen, Sweden. Samples: NEO052. Key contact: Catharina Ödman / Yvonne Magnusson. Institution: Malmö museum.
Vedbæk Boldbaner, Denmark. Samples: NEO600. Key contact: Niels Lynnerup, Anders Fischer, Pia Bennike. Institution: Sealand Archaeology/University of Copenhagen.
Vedrovice, Czech Republic. Samples: NEO128. Key contact: Vaclav Smrcka. Institution: Institute for History of Medicine and Foreign Languages of the First Faculty of Medicine, Charles University.
Vengerovo-2, Russia. Samples: NEO907, NEO910, NEO911, NEO912, NEO915, NEO916, NEO917, NEO918, NEO921, NEO922, NEO923. Key contact: V. I. Molodin. Institution: Institute of Archaeology and Ethnography, Russian Academy of Science, Siberian Branch.
Vibygårds Mose, Denmark. Samples: NEO935. Key contact: Anders Fischer / Per Lotz. Institution: Værløse Museum.
Viksø Mose, Denmark. Samples: NEO601. Key contact: Niels Lynnerup, Anders Fischer, Pia Bennike. Institution: Sealand Archaeology/University of Copenhagen.
Vittrup Mose, Denmark. Samples: NEO033. Key contact: Anders Fischer, Pia Bennike. Institution: Sealand Archaeology/University of Copenhagen.
Vlasac, Serbia. Samples: NEO655, NEO657, NEO658, NEO677. Key contact: Dušan Borić. Institution: Columbia university.
Volnensky, Ukraine. Samples: NEO300, NEO302, NEO304. Key contact: Sergey Vasilyev. Institution: Inst. of Ethnography and Anthropology, Moscow.
Voloshskoe, Ukraine. Samples: NEO522, NEO527. Key contact: Alexandra Buzhilova. Institution: Lomonosov Moscow State University.
Vovnigi, Ukraine. Samples: NEO498, NEO502, NEO503, NEO507, NEO508, NEO514, NEO524, NEO528, NEO529, NEO551, NEO552, NEO553. Key contact: Alexandra Buzhilova. Institution: Lomonosov Moscow State University.
Zamostje 2, Russia. Samples: NEO087, NEO088. Key contact: Alisa Zubova/Vyacheslav Moiseyev. Institution: Peter the Great Museum of Anthropology and ethnography (Kunstkamera).
Zhindo, Russia. Samples: NEO115, NEO117. Key contact: Sergey Vasilyev. Institution: Inst. of Ethnography and Anthropology, Moscow.

	Zvejnieki, Latvia. Samples: NEO307. Key contact: Sergey Vasilyev. Institution: Inst. of Ethnography and Anthropology, Moscow.
Specimen deposition	All specimens studied are available upon direct contact/request to the archaeologists, curators or officials responsible for their curation at the organisation where they are held.
Dating methods	272 novel radiocarbon dates were generated at the 14CHRONO laboratory, Queen's University Belfast (242 samples), at the Oxford Radiocarbon Accelerator Unit (ORAU) laboratory (24 samples) and at the Keck-CCAMS Group, Irvine, California, USA (6 samples).

☒ Tick this box to confirm that the raw and calibrated dates are available in the paper or in Supplementary Information.

Ethics oversight	No ethical oversight was required - all specimens were sampled following protocols designed to minimise invasive impact of sampling, with the full agreement of curators or archaeologists responsible for collections where specimens are held. Our use of shotgun whole genome data also reduces the need to resequence samples in the future (in contrast to targeting specific loci).

Note that full information on the approval of the study protocol must also be provided in the manuscript.
